# Effects of *Tetraselmis chuii* Microalgae Supplementation on Ergospirometric, Haematological and Biochemical Parameters in Amateur Soccer Players

**DOI:** 10.3390/ijerph17186885

**Published:** 2020-09-21

**Authors:** Víctor Toro, Jesús Siquier-Coll, Ignacio Bartolomé, María C. Robles-Gil, Javier Rodrigo, Marcos Maynar-Mariño

**Affiliations:** 1 Department of Physiology, School of Sport Sciences, University of Extremadura, University Avenue, s/n CP: 10003 Cáceres, Spain; vtororom@alumnos.unex.es (V.T.); ignbs.1991@gmail.com (I.B.); rodbelly@hotmail.com (J.R.); mmaynar@unex.es (M.M.-M.); 2 Department of Didactics of Musical, Plastic and Corporal Expression, School of Teacher Training, University of Extremadura, University Avenue, s/n CP: 10003 Cáceres, Spain; mcroblesgil@unex.es

**Keywords:** microalgae, soccer, ergogenic, ventilatory threshold

## Abstract

This study aimed to analyse the effects of *Tetraselmis chuii* (TC) microalgae supplementation during thirty days on ergospirometric, haematological and biochemical parameters in amateur soccer players. Thirty-two amateur soccer players divided into a control group (CG; *n* = 16; 22.36 ± 1.36 years; 68.36 ± 3.53 kg) and a supplemented group (SG; *n* = 16; 22.23 ± 2.19 years; 69.30 ± 5.56 kg) participated in the double-blind study. SG ingested 200 mg of the TC per day, while CG ingested 200 mg per day of lactose powder. Supplementation was carried out for thirty days. The participants performed a maximal treadmill test until exhaustion. The ergospirometric values at different ventilatory thresholds and haematological values were obtained after the test. Heart rate decreased after supplementation with TC (*p* < 0.05). Oxygen pulse, relative and absolute maximum oxygen consumption increased in SG (pre vs. post; 19.04 ± 2.53 vs. 22.08 ± 2.25; 53.56 ± 3.26 vs. 56.74 ± 3.43; 3.72 ± 0.35 vs. 3.99 ± 0.25; *p* < 0.05). Haemoglobin and mean corpuscular haemoglobin increased in SG (pre vs. post; 15.12 ± 0.87 vs. 16.58 ± 0.74 *p* < 0.01; 28.03 ± 1.57 vs. 30.82 ± 1.21; *p* < 0.05). On the other hand, haematocrit and mean platelet volume decreased in SG (*p* < 0.05). TC supplementation elicited improvements in ergospirometric and haematological values in amateur soccer players. TC supplementation could be valuable for improving performance in amateur athletes.

## 1. Introduction

Microalgae are photosynthetic eukaryotic microorganisms that live in the sea and were one of the first forms of life on Earth [1]. The number of microalgae species is estimated to range from 45,000 to more than 100,000 [2], and they have been used as a food source for humans for over a thousand years [3].

Microalgae can be considered a promising food due to their nutritional characteristics [4], and their industrial cultivation has increased in recent decades [5]. They have been used in the production of functional foods [6], animal feeds, biofuels [7] and cosmetics [8].

The effects of microalgae supplementation on humans have been studied previously [9,10], and marine bioactive peptides from microalgae have shown therapeutic potential in the treatment or prevention of disease [11]. The different effects of microalgae supplementation are anti-inflammatory [12], antioxidant [13], hypotensive [14] and hypolipidemic [15], among others. Supplementation with some microalgae such as spirulina and chlorella has been well studied [16,17]; however, the effect of supplementation with *Tetraselmis chuii* (TC) is unknown.

TC is green microalgae discovered in the 1950s, and is a unicellular, mobile, 4 to 15 μm size microseaweed, corresponding to the class Prasinophyceae [18]. It is present in the diet of mussels, oysters, clams, scallops and corals [19], representing a species of marine microalgae that is easy to grow and safe to eat [20]. Proportional to its size, TC has a high concentration of amino acids, essential fatty acids, vitamins and minerals (Table 1) [21,22].

The administration of sports supplements in soccer has become a standard procedure, often promoted by team physicians, coaches and even the parents of young players [23]. However, supplementation with microalgae is not common in sport, although their intake has been gradually introduced into sport due to the bioactive peptides they contain [24]. *Spirulina platensis* positively modifies bone marrow production and the cellular immune response, and may be effective as an adjuvant treatment in anaemia or immunodeficiency [25]. Similarly, other studies observed increases in maximum oxygen consumption (VO_2__max_) after supplementation with other microalgae, such as spirulina or chlorella [26,27]. Nevertheless, the literature about TC supplementation is scarce, and it would be interesting to know the impact of supplementation in humans due to its aforementioned properties. Therefore, this research aimed to evaluate the effects of TC microalgae supplementation during thirty days on ergospirometric parameters in amateur soccer players. In addition, the possible positive or negative effects on haematological and biochemical parameters were investigated.

## 2. Materials and Methods

### 2.1. Subjects

Thirty-two male players from a third division Spanish club participated in the study. The subjects were randomised into two groups: control group (CG; *n* = 16; 22.36 ± 1.36 years; 68.36 ± 3.53 kg; 1.74 ± 0.44 m) and supplemented group (SG; *n* = 16; 22.23 ± 2.19 years; 69.30 ± 5.56 kg; 1.73 ± 0.35 m). All participants were informed about the purpose of the study and signed a consent form before enrolling. The protocol was reviewed and approved by the Biomedical Ethics Committee of the University of Extremadura (Cáceres, Spain) following the guidelines of the Helsinki declaration of ethics, updated at the World Medical Assembly in Fortaleza (2013), for research involving human subjects (registration code: 99/2016). A code was assigned to each participant for the collection and treatment of the samples in order to maintain their anonymity. To be considered a healthy male and included in the study, participants had to comply with the inclusion criteria: not have haematological problems, not have altered values in the last blood analysis, not have anaemia problems, have four years of minimum training experience, be a nonsmoker, not have taken any supplementation, medication or over-the-counter medication, drug or alcohol in the previous two weeks and not to change their nutritional habits during the study.

At the beginning of the study, the participants completed a physical activity questionnaire (IPAQ) [28] and had a medical examination to detect any abnormalities. The participants performed 2.9 ± 0.55 metabolic equivalent of task (MET)-hour/day and no case abnormalities were reported.

### 2.2. Study Design

This research had a double-blind design. SG ingested a 200 mg capsule per day of powdered TC (TetraSOD^®^, El Puerto de Santa Maria, Andalucía, Spain) whereas CG ingested a 200 mg placebo tablet containing lactose powder. The nutritional value per day of the placebo pill was 22.6 kcal, 0.4 g water, 5.8 g carbohydrate, 0.1 g protein and 0.09 g lipids. Participants ingested the capsules for thirty days. Both capsules had identical designs to avoid interpretation among subjects. Participants were recommended to ingest all capsules at 10:00 a.m. for homogeneity of results. Table 1 shows the composition of TC [29].

The measurements were taken on the day previous to the beginning of the supplementation and after thirty days of supplementation. Participants did not intake TC on the day of the assessments. The assessments were performed after two days of inactivity to avoid the influence of training fatigue.

### 2.3. Blood Extraction and Determination of Haematological and Biochemical Parameters

Participants arrived between 8:00–9:00 a.m. for the extraction of 10 mL of venous blood from the antecubital vein. All extractions were carried out in fasting conditions. The blood sample was collected in a polypropylene tube. A 200 µL sample was taken from each blood tube and precipitated in a ladle and placed in the coulter (Coulter Electronics LTD, Model 6706319; Northwell Drive, Luton, UK) to obtain haematological data. For biochemical parameters, the blood was collected in 5 mL tubes containing ethylenediaminetetraacetic acid (EDTA) as anticoagulants and were centrifuged at 2500 rpm for 10 min. The plasma was separated and the biochemical parameters were determined by spectrophotometric techniques (Coulter Electronics LTD, Model CPA; Northwell Drive, Luton, UK).

### 2.4. Anthropometry

The anthropometric characteristics were measured in the morning at the same time after an overnight fast, always by the same researcher. A Seca^©^ 769 (Seca, Hamburg, Germany) scale, with an accuracy of ±100 g; a Seca^©^ 220 (Seca, Hamburg, Germany) measuring rod, accurate to ±1 mm; a Holtain^©^ 610ND (Holtain, Crymych, UK) skinfold compass, accurate to ±0.2 mm; a Holtain^©^ 604 (Holtain, Crymych, UK) bone diameter compass, accurate to ±1 mm; and a Seca^©^ 201 (Seca, Hamburg, Germany) brand tape measure, accurate to ±1 mm, were used for the anthropometric assessments. The equations of the Spanish Group of Kinanthropometry [30] were used to calculate the muscle, fat and bone percentage. The anthropometric measurements obtained were height, weight, skinfolds (abdominal, suprailiac, subscapular, tricipital, thigh and leg), bone diameters (bistyloid, humeral biepicondyle and femoral biepicondyle) and muscle perimeters (relaxed arm and leg).

### 2.5. Nutritional Evaluation

All participants completed a nutritional survey in the first and last week of the study to guarantee that they were following a similar diet. The survey consisted of a 4-day daily nutritional record, of three preassigned week days, and one weekend day. The participants individually indicated the type, frequency and quantity (in grams) of each food consumed each day, and the nutritional composition of their diets was evaluated using different food composition tables [31].

### 2.6. Maximum Incremental Test and Threshold Determination

Subjects performed an incremental ergospirometric test on a treadmill (Ergofit Trac Alpin 4000, Germany). The test started with a warm-up at 8 km/h for 10 min and was increased by 1 km/h every two minutes until voluntary exhaustion. The tests were carried out in the laboratory with ambient conditions of 23 ± 2 °C (45–55% relative humidity). Physiological ergospirometric parameters were monitored with a gas analyser (Metamax model no. 762014-102, Cortex, Germany), and heart rate (HR) was monitored with a pulsometer (Polar^®^ “Vantage M”, Kempele, Finland) with sensor band (Polar^®^ H10, Kempele, Finland). All tests were performed from 11:30 a.m. onwards in the same order to avoid the effects of circadian cycles.

After recording the test data, the ventilatory thresholds were determined according to the three-phase model [32]. The data were obtained at the aerobic threshold (VT1), the anaerobic threshold (VT2), maximum values of the incremental test and after three minutes of recovery.

### 2.7. Statistical Analysis

Statistical analyses were performed with SPSS 20.0 for Windows (SPSS Inc., Chicago, IL, USA). The normality of the distribution of variables was analysed using the Shapiro–Wilk test and the homogeneity of the variances with the Levene test. A paired samples t-test was used to compare the differences between pre- and post-supplementation and an independent samples t-test was used to compare the differences between CG and SG. A *p* < 0.05 was considered statistically significant. Results are expressed as means ± standard deviation.

## 3. Results

The results obtained are presented below, before supplementation (pre) and after supplementation (post). Table 2 shows the anthropometric values in both groups. No significant changes were observed between groups.

Table 3 shows the intake of macronutrients in both groups. There was no significant difference in total macronutrient intake.

Table 4 shows the ergospirometric data of VT1 in both groups. There was a significant decrease in HR in the SG after TC supplementation (*p* < 0.05). There were no significant differences in the other parameters.

Table 5 shows the results obtained for both groups at VT2. A significant decrease can be observed in HR in SG after TC supplementation compared to CG and baseline values (*p* < 0.01). There were no significant differences in the remaining parameters.

Table 6 shows the maximum values obtained in the incremental test. Decreases in HRmax were observed after TC supplementation in SG compared to baseline (*p* < 0.05) and CG (*p* < 0.05). In SG, there were significant increases in oxygen pulse, and maximum absolute and relative oxygen consumption (VO_2_) values (*p* < 0.05).

**Figure 1:**
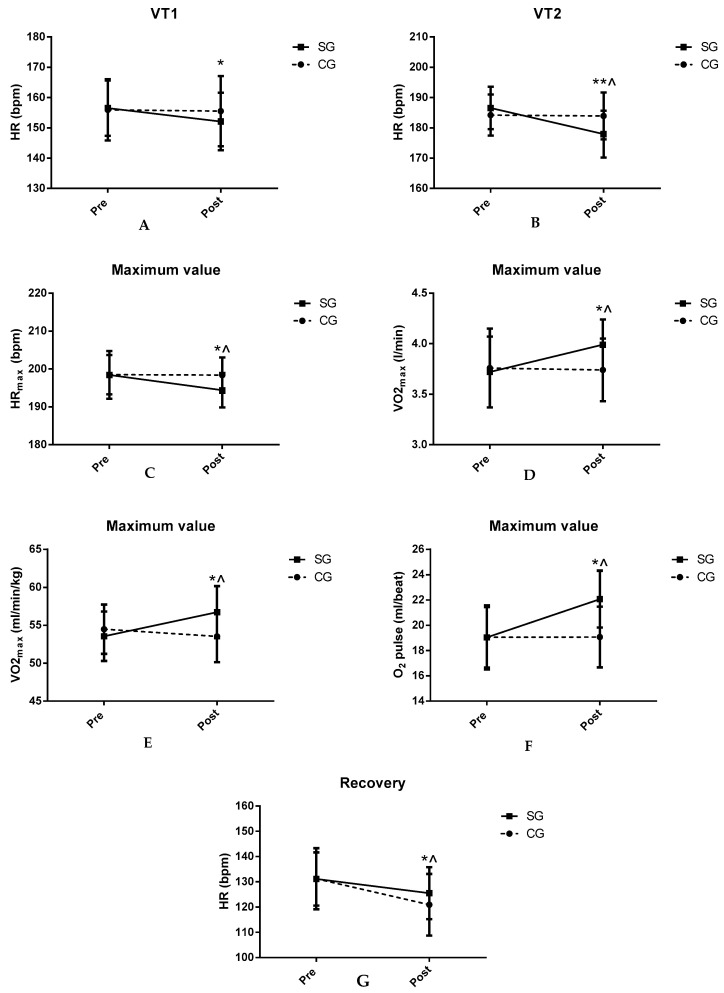
Results of the incremental test: (**A**) ergospirometric values corresponding to VT1; (**B**) ergospirometric values corresponding to VT2; (**C**–**F**) maximum values of the incremental test; (**G**) results obtained after three minutes of recovery. CG: control group; SG: supplemented group; HR: heart rate; VO_2_: oxygen consumption; * *p* < 0.05 differences pre vs. post in SG; ** *p* < 0.01 differences pre vs. post in SG; ^ *p* < 0.05 differences post CG vs. post SG.

Table 7 shows the ergospirometric values obtained after 3 min of recovery at the end of the maximum incremental test. Significant decreases in HR in SG were found after TC supplementation compared to CG and baseline values (*p* < 0.05). There were no significant differences in the other parameters. Figure 1 shows the key findings.

Table 8 shows the biochemical parameters studied in both groups. There were increases in glucose (*p* < 0.01), uric acid (*p* < 0.05) and creatinine (*p* < 0.01) values after TC supplementation in SG. In addition, the previous parameters after TC supplementation were higher in SG compared to CG.

Table 9 shows the values obtained for the different haematological parameters studied in both groups. There were increases in haemoglobin (*p* < 0.01) and mean corpuscular haemoglobin (MCH) (*p* < 0.05) values after TC supplementation in SG compared to CG and baseline values. Haematocrit values and MPV decreased in SG after TC supplementation (*p* < 0.05).

## 4. Discussion

The aim of the present study was to analyse the effect of TC (TetraSOD^®^ El Puerto de Santa Maria, Andalucía, Spain) supplementation on ergospirometric, haematological and biochemical parameters in amateur soccer players. To our knowledge, this is the first study to evaluate the impact of TC supplementation in athletes. TC supplementation during thirty days produced decreases in HR and increased absolute VO_2__max_, relative VO_2__max_, oxygen pulse, haemoglobin and mean corpuscular haemoglobin (MCH) (*p* < 0.05) In addition, glucose, uric acid and creatinine were higher in SG. No changes were observed in CG, which could indicate that the changes in SG could be due to TC supplementation. It should be noted that there were no differences in nutritional intake. All biochemical parameters were maintained in normal ranges. No negative effects on the body were observed.

TetraSOD^®^ is a unique commercial product composed of 100% lyophilised TC that is currently marketed for food and nutraceutical applications. In 2017, the European Union gave approval to the company to market its lyophilised TC for use in dietary supplements such as TetraSOD^®^, at levels of up to 200 mg/day [20].

The decrease of HR in SG could be positively related to arterial stiffness [33,34]. Previous studies have investigated the effects of the supplementation of other green microalgae, the microalgae chlorella, on arterial stiffness. Otsuki et al. (2013) analysed the effect of 200 mg chlorella supplementation in fourteen men over twelve weeks in a double-blind trial [35]. They concluded that chlorella supplementation could decrease arterial stiffness due to the nutrients it contains. Another study analysed the supplementation of 200 mg of chlorella in thirty-two subjects, with the authors concluding that multicomponent supplementation derived from chlorella decreased arterial stiffness [36]. Thus, it could be assumed that differences in HR may be due to changes in arterial stiffness; when artery walls lose their elastic properties and become stiff, they elicit a rise in systolic blood pressure and the heart’s workload [37].

Some components present in TC may positively affect arterial stiffness; minerals such as potassium could decrease it [38], as well as unsaturated fatty acids through their anti-inflammatory function [39]. According to the results obtained, TC could reduce arterial stiffness due to the nutrients it contains and, therefore, decrease HR. The decrease in HR is related to the increase in O_2_ pulse [40]. This could indicate a better metabolic economy of effort by the body.

Concerning ergospirometric values, SG recorded an increase in absolute and relative VO_2__max_ (*p* < 0.05). Other research has analysed the effect of supplementation with other microalgae in different groups. For example, Hernández-Lepe et al. (2018) evaluated an exercise programme with and without spirulina, and a nonexercise programme with and without spirulina, in sedentary overweight and obese people [26]. They observed that supplementation of 4.5 g/day for six weeks with spirulina increased the relative VO_2__max_ in the groups that consumed the microalgae without the exercise programme. Additionally, they reported a decrease in resting HR and an increase in the maximum lactate steady state. Another crossover and double-blind study reported a rise in relative VO_2__max_ after twice-daily supplementation with chlorella (200 mg) for four weeks in ten young people [41]. In a double-blind trial by Zempo-Miyaki et al. (2017), a multicomponent supplement derived from chlorella (200 mg) for four weeks in thirty-four healthy men increased relative VO_2__max_ in an incremental maximal test on a cycle ergometer [27].

Curiously, the maximal speed in the test before and after the supplementation in SG was similar, whereas there was an increase in absolute and relative VO_2__max_. The rise in VO_2_ could be due to an increase in O_2_ pulse and haemoglobin. The calcium content of TC could generate an increase in oxygen transport by the haemoglobin. Calcium performs several vital functions in red blood cells that affect oxygen transport and coagulation, and increase the cell half-life [42,43]. Increases in intracellular calcium appear to promote the ability of red blood cells to supply oxygen [43].

TC contains carotenoids and polyphenols [20] that have antioxidant properties [44]. Several investigations observed that spirulina supplementation increases some enzymatic antioxidant systems [45,46]. The antioxidant properties of TC could explain the performance increase observed in this study.

Haemoglobin and MCH concentrations increased after TC supplementation in SG (*p* < 0.01 in haemoglobin; *p* < 0.05 in MCH). Microalgae have been used in animals as supplements to improve iron deficiencies [47]. Nasirian et al. (2017) investigated the effects of *Spirulina platensis* (15 and 30 mg/kg body weight) for five weeks on haematological parameters in diabetic rats [48]. They observed that *Spirulina platensis* supplementation of 30 mg/kg body weight improved total red blood cells, haemoglobin, MCH, mean cell volume and whole white blood cells. The authors hypothesised that *Spirulina platensis* could stimulate erythropoietin formation. In humans, Selmi et al. (2011) analysed the effect of 500 mg spirulina supplementation for twelve weeks in forty older volunteers [49]. They reported an increase in MCH in subjects of both sexes, whereas MCV and MCH concentration increased in males. They concluded that more studies are needed to confirm the results in humans. It should be noted that the participants in the previous studies were animals and older people, a different population to those of the present study.

The increases in haemoglobin concentration in SG despite a drop in haematocrit are discordant. This phenomenon could be related to the presence of some haematopoietic factor in the algae. The chlorides present in the microalgae could explain this increase [9], as cobalt chloride is an erythropoietic factor [50]. Another mineral, such as iron, could explain this increase in haemoglobin. The effects of iron on erythropoiesis and haemoglobin levels have been previously reported [51]. Finally, the bioactive peptides present in the microalgae could play a significant role as they have immunomodulation, antihypertensive or anticancer properties [52]. We believe that during the TC digestion process some bioactive peptide with haematopoietic properties can be obtained. In addition, we believe that the high levels of sodium and chloride present in TC could increase fluid retention and generate hypervolaemia, thereby decreasing haematocrit [53,54]. Further research is needed to explain the results obtained. We think that it is a finding of interest for sports performance.

This study has many limitations as it is a preliminary study of algae, which at this moment has not been previously investigated. First, the intensity and volume of training during the study could influence the results. The team’s coach confirmed that the number of training hours and days did not change during the study in both groups. However, the intensity could have been modified. Second, the number of participants in this study was small and micronutrients intake was not considered. Third, there are no studies on these microalgae to compare results, so studies on other algae have been used. Fourth, the state of hydration could not be evaluated to verify the increase in plasma volume.

Current results need to be confirmed with larger sample sizes, and different populations and concentrations of TC to analyse the potential effects of various doses, and establish a dose–response relationship. It would be interesting for future studies to investigate the antioxidant properties of TC as well as the effect on elite athletes, and analyse the intake of micronutrients.

## 5. Conclusions

The data obtained in this investigation suggest that the daily supplementation of 200 mg of TC (TetraSOD^®^) during thirty days could modify the ergospirometric and haematological parameters analysed in amateur soccer players. The absence of abnormal values in biochemical parameters would indicate that the TC microalgae would not have negative effects on our body. Although these are preliminary results, we suggest that TC could be an ergogenic alternative for athletes. The intake of TC significantly improved the values of the hemogram, which would probably be the cause of the ergospirometric changes. The reasons for ergospirometric and haematological changes after TC administration are not well understood, and further research is needed to elucidate this topic.

## Figures and Tables

**Table 1:** Composition of TC from Fitoplacton Marino S.L.

Component	Quantity	Component	Quantity
Proteins (mg/pill)	75.2 ± 2.51	Calcium (mg/g)	33.8 ± 0.26
Carbohydrates (mg/pill)	63.2 ± 2.67	Phosphorus (mg/g)	6.27 ± 1.87
Lipids (mg/pill)	13.4 ± 1.04	Magnesium (mg/g)	5.06 ± 0.09
Saturated fatty acids (mg/pill)	4.06 ± 0.41	Potassium (mg/g)	10.40 ± 0.56
Monounsaturated fatty acids (mg/pill)	7.05 ± 0.86	Sodium (mg/g)	14.33 ± 4.16
Polyunsaturated fatty acids (mg/pill)	6.26 ± 0.77	Chloride (mg/g)	17.77 ± 0.25
Leucine (mg/pill)	1.15 ± 0.08	Copper (mg/g)	0.006 ± 0.0
Arginine (mg/pill)	1.00 ± 0.06	Iron (mg/g)	2.01 ± 0.01
Glutamic acid (mg/pill)	1.75 ± 0.08	Manganese (mg/g)	5.06 ± 0.09
Aspartic acid (mg/pill)	1.39 ± 0.06	Iodine (mg/kg)	5.03 ± 5.78

**Table 2:** Anthropometric values.

	CG (*n* = 16)			SG (*n* = 16)		
	Pre	Post	*p* Value	Pre	Post	*p* Value
Total weight (kg)	69.55 ± 4.70	69.10 ± 5.80	0.623	69.74 ± 5.89	69.05 ± 5.80	0.576
Σ6 skinfolds	68.00 ± 18.21	68.12 ± 17.95	0.891	68.38 ± 18.67	67.54 ± 18.05	0.401
Muscle percentage	48.39 ± 1.70	48.45 ± 2.67	0.678	48.48 ± 1.65	49.24 ± 2.28	0.245
Bone percentage	17.15 ± 0.83	17.17 ± 1.70	0.891	17.10 ± 0.87	17.15 ± 0.72	0.813
Fat percentage	10.41 ± 1.60	10.37 ± 1.70	0.451	10.30 ± 1.79	9.80 ± 1.39	0.378

CG: control group; SG: supplemented group; Σ: summation.

**Table 3:** Nutritional assessment in the first and the last weeks of the study.

	CG (*n* = 16)			SG (*n* = 16)		
	Pre	Post	*p* Value	Pre	Post	*p* Value
Total intake (kcal/days)	2304.31 ± 321.21	2266.25 ± 307.27	0.556	2368.63 ± 347.41	2286.47 ± 339.74	0.347
Carbohydrates (g/days)	281.35 ± 35.24	277.43 ± 31.43	0.469	285.41 ± 28.31	279.51 ± 30.02	0.338
Proteins (g/days)	128.28 ± 25.62	130.84 ± 22.35	0.629	127.47 ± 26.67	130.32 ± 25.72	0.478
Lipids (g/days)	88.24 ± 21.24	85.45 ± 22.37	0.437	85.39 ± 26.81	82.25 ± 22.45	0.405

g: grams; kcal: kilocalories.

**Table 4:** Ergospirometric values corresponding to VT1.

	CG (*n* = 16)			SG (*n* = 16)		
	Pre	Post	*p* Value	Pre	Post	*p* Value
Speed (km/h)	10.55 ± 0.69	10.62 ± 1.12	0.567	10.71 ± 0.73	10.70 ± 1.09	0.897
HR (bpm)	155.95 ± 10.1	155.50 ± 11.55	0.821	156.50 ± 9.10	152.10 ± 9.51 *	0.041
Absolute VO_2_ (L/min)	2.46 ± 0.22	2.47 ± 0.20	0.789	2.47 ± 0.30	2.48 ± 0.27	0.702
Relative VO_2_ (mL/min/kg)	35.40 ± 2.50	35.80 ± 3.01	0.643	35.51 ± 2.87	36.59 ± 3.11	0.418
VCO_2_ (L/min)	2.20 ± 0.25	2.22 ± 0.31	0.609	2.23 ± 0.26	2.21 ± 0.30	0.587
RER (VCO_2_/VO_2_)	0.90 ± 0.02	0.90 ± 0.01	0.803	0.90 ± 0.02	0.90 ± 0.03	0.789
O_2_ pulse (mL/beat)	15.90 ± 2.09	15.99 ± 1.10	0.495	15.93 ± 2.10	16.05 ± 1.04	0.521
VE (L/min)	68.30 ± 11.05	68.52 ± 11.01	0.613	68.29 ± 11.38	69.50 ± 11.09	0.406
VE/VO_2_	25.50 ± 2.36	25.55 ± 2.22	0.872	25.64 ± 2.76	25.77 ± 2.29	0.621
VE/VCO_2_	28.59 ± 2.64	28.60 ± 2.13	0.883	28.60 ± 2.74	28.61 ± 2.04	0.813

CG: control group; SG: supplemented group; HR: heart rate; VO_2_: oxygen consumption; VCO_2_: carbon dioxide consumption; RER: respiratory exchange ratio; O_2_: oxygen; VE: expired volume; *p* value: differences pre vs. post; *: p < 0.05 differences pre vs. post in SG.

**Table 5:** Ergospirometric values at VT2.

	CG (*n* = 16)			SG (*n* = 16)		
	Pre	Post	*p* Value	Pre	Post	*p* Value
Speed (km/h)	15.45 ± 1.32	15.10 ± 1.12	0.701	15.50 ± 1.02	15.87 ± 1.34	0.831
HR (bpm)	184.23 ± 6.80	183.95 ± 7.75	0.696	186.57 ± 7.00	177.92 ± 7.75 ^	0.008
Absolute VO_2_ (L/min)	3.33 ± 0.41	3.34 ± 0.24	0.845	3.34 ± 0.43	3.37 ± 0.36	0.439
Relative VO_2_ (mL/min/kg)	47.60 ± 3.65	47.98 ± 3.56	0.793	47.71 ± 3.97	48.36 ± 3.26	0.278
VCO_2_ (L/min)	3.40 ± 0.42	3.39 ± 0.30	0.868	3.41 ± 0.41	3.40 ± 0.27	0.512
RER (VCO_2_/VO_2_)	1.02 ± 0.01	1.01 ± 0.01	0.771	1.02 ± 0.02	1.01 ± 0.01	0.814
O_2_ pulse (mL/beat)	18.49 ± 1.20	18.51 ± 1.28	0.882	18.52 ± 1.80	18.60 ± 1.26	0.708
VE (L/min)	108.34 ± 9.43	107.10 ± 9.90	0.791	108.14 ± 9.48	106.50 ± 10.00	0.451
VE/VO_2_	29.80 ± 2.41	29.73 ± 2.48	0.702	29.83 ± 2.47	29.69 ± 2.58	0.512
VE/VCO_2_	29.27 ± 2.29	29.45 ± 2.45	0.845	29.24 ± 2.23	29.49 ± 2.55	0.689

CG: control group; SG: supplemented group; HR: heart rate; VO_2_: oxygen consumption; VCO_2_: carbon dioxide consumption; RER: respiratory exchange ratio; O_2_: oxygen; VE: expired volume; *p* value: differences pre vs. post; ^: *p* < 0.05 differences post CG vs. post SG.

**Table 6:** Maximum values of the incremental test in both groups.

	CG (*n* = 16)			SG (*n* = 16)		
	Pre	Post	*p* Value	Pre	Post	*p* Value
Speed (km/h)	19.60 ± 0.89	19.58 ± 0.92	0.834	19.64 ± 0.93	19.82 ± 0.90	0.603
HR_max_ (bpm)	198.50 ± 5.18	198.38 ± 4.63	0.781	198.43 ± 6.28	194.42 ± 4.52 ^	0.046
Absolute VO_2__max_ (L/min)	3.76 ± 0.39	3.74 ± 0.31	0.491	3.72 ± 0.35	3.99 ± 0.25 ^	0.031
Relative VO_2__max_ (mL/min/kg)	54.49 ± 3.25	53.55 ± 3.40	0.309	53.56 ± 3.26	56.74 ± 3.43 ^	0.043
VCO_2_ (L/min)	4.34 ± 0.40	4.35 ± 0.39	0.843	4.35 ± 0.59	4.32 ± 0.41	0.418
RER (VCO_2_/VO_2_)	1.17 ± 0.04	1.16 ± 0.03	0.619	1.17 ± 0.05	1.16 ± 0.04	0.626
O_2_ pulse (mL/beat)	19.06 ± 2.40	19.08 ± 2.41	0.792	19.04 ± 2.53	22.08 ± 2.25 ^	0.045
VE (L/min)	144.00 ± 17.21	144.01 ± 18.98	0.925	142.14 ± 18.2	146.50 ± 19.35	0.221
VE/VO_2_	36.50 ± 4.11	37.00 ± 4.09	0.213	36.63 ± 4.57	37.52 ± 4.02	0.426
VE/VCO_2_	31.61 ± 2.23	31.98 ± 2.32	0.678	31.68 ± 2.73	32.49 ± 2.85	0.406

CG: control group; SG: supplemented group; HRmax: maximum heart rate; VO_2__max_: maximum oxygen consumption; VCO_2_: carbon dioxide consumption; RER: respiratory exchange ratio; O_2_: oxygen; VE: expired volume; *p* value: differences pre vs. post; ^: *p* < 0.05 differences post CG vs. post SG.

**Table 7:** Ergospirometric results obtained after three minutes of recovery.

	CG (*n* = 16)			SG (*n* = 16)		
	Pre	Post	*p* Value	Pre	Post	*p* Value
HR (bpm)	131.23 ± 12.12	130.90 ± 12.20	0.705	131.10 ± 10.51	125.50 ± 10.29 ^	0.039
VO_2_ (L/min)	0.95 ± 0.38	0.94 ± 0.19	0.645	0.96 ± 0.28	0.95 ± 0.36	0.715
Relative VO_2_/kg (mL/min/kg)	13.29 ± 2.45	13.01 ± 2.15	0.479	13.57 ± 3.05	14.08 ± 2.05	0.255
Absolute VCO_2_ (L/min)	1.20 ± 0.37	1.19 ± 0.25	0.631	1.22 ± 0.38	1.20 ± 0.20	0.521
RER (VCO_2_/VO_2_)	1.28 ± 0.10	1.29 ± 0.09	0.699	1.29 ± 0.11	1.28 ± 0.07	0.589
O_2_ pulse (mL/beat)	7.32 ± 1.32	7.11 ± 1.01	0.306	7.38 ± 2.35	7.17 ± 0.94	0.201
VE (L/min)	46.90 ± 14.51	45.11 ± 12.36	0.218	46.14 ± 15.57	45.07 ± 13.40	0.349
VE/VO_2_	45.40 ± 5.97	45.32 ± 5.68	0.521	45.58 ± 6.88	44.86 ± 5.08	0.180
VE/VCO_2_	35.28 ± 3.70	35.26 ± 3.57	0.844	35.34 ± 3.83	35.31 ± 3.47	0.719

CG: control group; SG: supplemented group; HR: heart rate; VO_2_: oxygen consumption; VCO_2_: carbon dioxide consumption; RER: respiratory exchange ratio; O_2_: oxygen; VE: expired volume; *p* value: differences pre vs. post; ^: *p* < 0.05 differences post CG vs. post SG.

**Table 8:** Biochemical values of both groups.

	CG (*n* = 16)			SG (*n* = 16)		
	Pre	Post	*p* Value	Pre	Post	*p* Value
Glucose (mg/dL)	84.02 ± 8.29	85.10 ± 6.30	0.417	83.01 ± 10.31	91.01 ± 5.40 ^	0.007
Cholesterol (mg/dL)	113.11 ± 20.81	110.78 ± 25.15	0.327	112.27 ± 22.73	107.78 ± 24.40	0.219
Triglycerides (mg/dL)	51.44 ± 26.20	49.60 ± 20.30	0.415	50.79 ± 35.39	43.59 ± 19.07	0.105
Uric acid (mg/dL)	5.74 ± 0.72	5.75 ± 0.73	0.735	5.76 ± 0.76	6.04 ± 0.77 ^	0.035
HDL (mg/dL)	52.02 ± 6.50	53.60 ± 7.61	0.621	51.14 ± 6.35	55.54 ± 7.50	0.195
LDL (mg/dL)	50.37 ± 20.21	49.12 ± 20.11	0.569	50.97 ± 20.62	43.58 ± 20.91	0.077
GOT (U/L)	36.23 ± 13.76	36.11 ± 12.93	0.877	36.79 ± 14.91	36.06 ± 11.96	0.421
GPT (U/L)	27.45 ± 14.12	26.88 ± 12.31	0.521	27.32 ± 15.12	25.88 ± 10.38	0.451
GGT (U/L)	21.40 ± 18.06	22.02 ± 17.21	0.610	21.53 ± 19.07	22.25 ± 17.46	0.653
Creatinine (mg/dL)	0.96 ± 0.11	0.97 ± 0.10	0.690	0.96 ± 0.10	1.07 ± 0.12 ^	0.043

CG: control group; SG: supplemented group; HDL: high density lipoprotein; LDL: low density lipoprotein; GOT: glutamic-oxaloacetic transaminase; GPT: glutamic-pyruvic transaminase; GGT: gamma-glutamyl transferase; *p* value: differences pre vs. post; ^: *p* < 0.05 differences post CG vs. post SG.

**Table 9:** Haemogram of both groups in the different evaluations.

	CG (*n* = 16)			SG (*n* = 16)		
	Pre	Post	*p* Value	Pre	Post	*p* Value
Red blood cells (millions)	5.40 ± 0.31	5.39 ± 0.35	0.734	5.43 ± 0.34	5.42 ± 0.39	0.569
Haemoglobin (gr %)	15.25 ± 0.80	15.24 ± 0.84	0.459	15.12 ± 0.87	16.58 ± 0.74 **^	0.008
Haematocrit (%)	48.29 ± 2.72	48.45 ± 2.15	0.743	48.03 ± 2.15	46.68 ± 2.05 *^	0.045
MCV (fL)	88.00 ± 2.55	87.84 ± 3.08	0.585	88.10 ± 2.75	87.49 ± 4.05	0.418
MCH (Pg)	28.31 ± 1.60	29.26 ± 1.69	0.476	28.03 ± 1.57	30.82 ± 1.21 *	0.042
Platelets (thousands)	223.67 ± 70.05	225.39 ± 65.56	0.702	222.72 ± 90.05	241.31 ± 46.99	0.216
MPV (fL)	9.00 ± 1.42	9.01 ± 1.20	0.833	9.02 ± 1.13	8.04 ± 1.05 *^	0.041
Leukocytes (thousands)	6.57 ± 1.17	6.53 ± 1.73	0.704	6.55 ± 1.67	6.51 ± 1.73	0.491
Lymphocytes (10^3^/μL)	1.90 ± 0.60	1.89 ± 0.33	0.755	1.89 ± 0.61	1.88 ± 0.42	0.306
Neutrophils (10^3^/μL)	3.78 ± 1.10	3.75 ± 1.12	0.649	3.77 ± 1.24	3.76 ± 1.09	0.703
Monocytes (10^3^/μL)	0.40 ± 0.12	0.41 ± 0.20	0.805	0.39 ± 0.15	0.41 ± 0.24	0.498
Basophils (10^3^/μL)	0.05 ± 0.02	0.06 ± 0.05	0.876	0.05 ± 0.03	0.06 ± 0.06	0.614
Eosinophil (10^3^/μL)	0.28 ± 0.18	0.27 ± 0.19	0.859	0.28 ± 0.19	0.26 ± 0.20	0.592
ESR (mm)	6.59 ± 2.80	6.41 ± 2.90	0.621	6.53 ± 3.61	6.43 ± 2.73	0.217

CG: control group; SG: supplemented group; MCV: medium corpuscular volume; MCH: mean corpuscular haemoglobin; MPV: mean platelet volume; ESR: erythrocyte sedimentation rate; *p* value: differences pre vs. post; ^ *p* < 0.05 differences post CG vs. post SG.
